# Machine learning model for identifying important clinical features for predicting remission in patients with rheumatoid arthritis treated with biologics

**DOI:** 10.1186/s13075-021-02567-y

**Published:** 2021-07-06

**Authors:** Bon San Koo, Seongho Eun, Kichul Shin, Hyemin Yoon, Chaelin Hong, Do-Hoon Kim, Seokchan Hong, Yong-Gil Kim, Chang-Keun Lee, Bin Yoo, Ji Seon Oh

**Affiliations:** 1 Department of Internal Medicine, Inje University Seoul Paik Hospital, Inje University College of Medicine, Seoul, Korea; 2 Department of Management Engineering, College of Business, KAIST, Seoul, Korea; 3 Division of Rheumatology, Seoul Metropolitan Government-Seoul National University Hospital Boramae Medical Center, Seoul, Korea; 4 Big Data Research Center, Asan Institute of Life Sciences, Asan Medical Center, Seoul, Korea; 5 Department of Information Medicine, Asan Medical Center, 88, Olympic-ro 43-gil, Songpa-gu, Seoul, Korea; 6 Division of Rheumatology, Department of Internal Medicine, Asan Medical Center, University of Ulsan College of Medicine, Seoul, Korea

**Keywords:** Rheumatoid arthritis, Biologics, Remission, Machine learning, Explainable artificial intelligence

## Abstract

**Background:**

We developed a model to predict remissions in patients treated with biologic disease-modifying anti-rheumatic drugs (bDMARDs) and to identify important clinical features associated with remission using explainable artificial intelligence (XAI).

**Methods:**

We gathered the follow-up data of 1204 patients treated with bDMARDs (etanercept, adalimumab, golimumab, infliximab, abatacept, and tocilizumab) from the Korean College of Rheumatology Biologics and Targeted Therapy Registry. Remission was predicted at 1-year follow-up using baseline clinical data obtained at the time of enrollment. Machine learning methods (e.g., lasso, ridge, support vector machine, random forest, and XGBoost) were used for the predictions. The Shapley additive explanation (SHAP) value was used for interpretability of the predictions.

**Results:**

The ranges for accuracy and area under the receiver operating characteristic of the newly developed machine learning model for predicting remission were 52.8–72.9% and 0.511–0.694, respectively. The Shapley plot in XAI showed that the impacts of the variables on predicting remission differed for each bDMARD. The most important features were age for adalimumab, rheumatoid factor for etanercept, erythrocyte sedimentation rate for infliximab and golimumab, disease duration for abatacept, and C-reactive protein for tocilizumab, with mean SHAP values of − 0.250, − 0.234, − 0.514, − 0.227, − 0.804, and 0.135, respectively.

**Conclusions:**

Our proposed machine learning model successfully identified clinical features that were predictive of remission in each of the bDMARDs. This approach may be useful for improving treatment outcomes by identifying clinical information related to remissions in patients with rheumatoid arthritis.

**Supplementary Information:**

The online version contains supplementary material available at 10.1186/s13075-021-02567-y.

## Introduction

Rheumatoid arthritis (RA) is a chronic inflammatory disease that affects the synovial tissues in multiple joints. Biologics are often considered a promising line of treatment for patients who have high disease activities despite treatment with conventional disease-modifying anti-rheumatic drugs (cDMARDs) [1–3]. Biologics are generally prescribed by referring to clinical practice recommendations after considering factors such as disease activity, adverse events, and cost effectiveness [1–3]. However, treatment with biologics may be unsuccessful because of differences in the physiological and pathological characteristics among individuals. Indeed, clinical trials have frequently shown that approximately 30–40% of patients do not respond to treatment with biologics, and their response rates decrease with subsequent biologics [4, 5]. Treatment failure due to ineffective biologics not only increases the pain experienced by the individual but also increases their cost of healthcare [6]. Therefore, it is necessary to develop good predictors that can identify the efficacies of different biologics for such individuals.

Several clinical, genetic, and proteomic studies have used statistical methods to identify biomarkers to predict responses to biologics in patients with RA [7–9]. Machine learning approaches that can complement statistical methods are able to incorporate such information for making accurate predictions. Furthermore, machine learning can be generalized across a broader array of data types and can produce results with complex situations as well [10, 11]. However, it is often difficult for users to understand the processes by which machine learning predicts outcomes from relationships among numerous variables. Accordingly, several methods have been proposed to improve the interpretability of machine learning methods while maintaining the prediction accuracies of complex models. Explainable artificial intelligence (XAI), which presents the reasons for a prediction in a manner that can be understood, suggests the relationships between several variables necessary for predicting outcomes [12].

In the field of artificial intelligence, XAI was recently developed to help our understanding of the important features that are related to predicting the outcomes of machine learning models. In the study of RA with various clinical characteristics, it is possible to use the machine learning method to predict outcomes such as remission and show important clinical features that are associated with the desired outcomes. Therefore, predicting remission with a machine learning model by combining multiple variables in RA cohort data and identifying important features associated with remission by using XAI is an advanced approach that complements the traditional statistical methods for determining the relationship between remission and various variables.

In this study, we established a machine learning model using data from the Korean College of Rheumatology Biologics and Targeted Therapy Registry (KOBIO) [13] and show its application to identifying clinical variables predictive of remission in RA patients treated with biologics using the concept of XAI.

## Materials and methods

### Study population

This study used data from the KOBIO registry, which is a nationwide multicenter cohort in Korea that was established to evaluate the effectiveness and side effects of biologic therapies in patients with RA [13]. Patients in the registry were recruited from 38 hospitals since 2012, and their demographics, medications, comorbidities, extra-articular manifestations, disease activities, radiographic findings, and laboratory findings performed within 4 weeks prior to the patient’s visit were recorded with the date. The data from patients who were followed up annually were recorded on the KOBIO website (http://www.kobio.or.kr/kobio/), and these patients provided informed consent prior to registration. Ethical approval of the KOBIO-RA was obtained from the institutional review boards of all 38 participating institutions, including the Institutional Review Board of Inje University Seoul Paik Hospital (PAIK 2018-11-005).

### Data collection

**Fig. 1:**
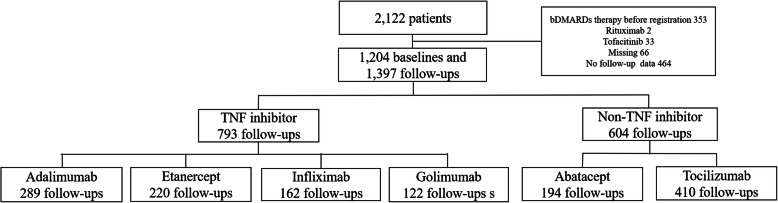
Flowchart of patient selection. bDMARDs, biologic disease-modifying anti-rheumatic drugs; TNF, tumor necrosis factor

Figure 1 shows the flowchart for patient selection. From December 2012 to June 2019, a total of 2122 patients who were treated with biologic disease-modifying anti-rheumatic drugs (bDMARDs) and targeted synthetic disease-modifying anti-rheumatic drugs (tsDMARDs) were registered. The baseline data were obtained at the time of initial enrollment for prescription of the bDMARDs, and follow-up data were registered annually or when the bDMARDs were switched or stopped. Patients treated with tsDMARDs, such as tofacitinib (N = 33), were excluded from the analysis because the aim of the study was to predict patients who responded to bDMARDs. Among the bDMARDs, rituximab was excluded from the analysis owing to the small sample group (N = 2). After excluding data from subjects whose follow-up durations were less than 3 months from the baseline, a total of 1204 baseline data and 1397 follow-up data were obtained and used for the analysis in this study.

### Machine learning methodology for predicting remission

**Fig. 2:**
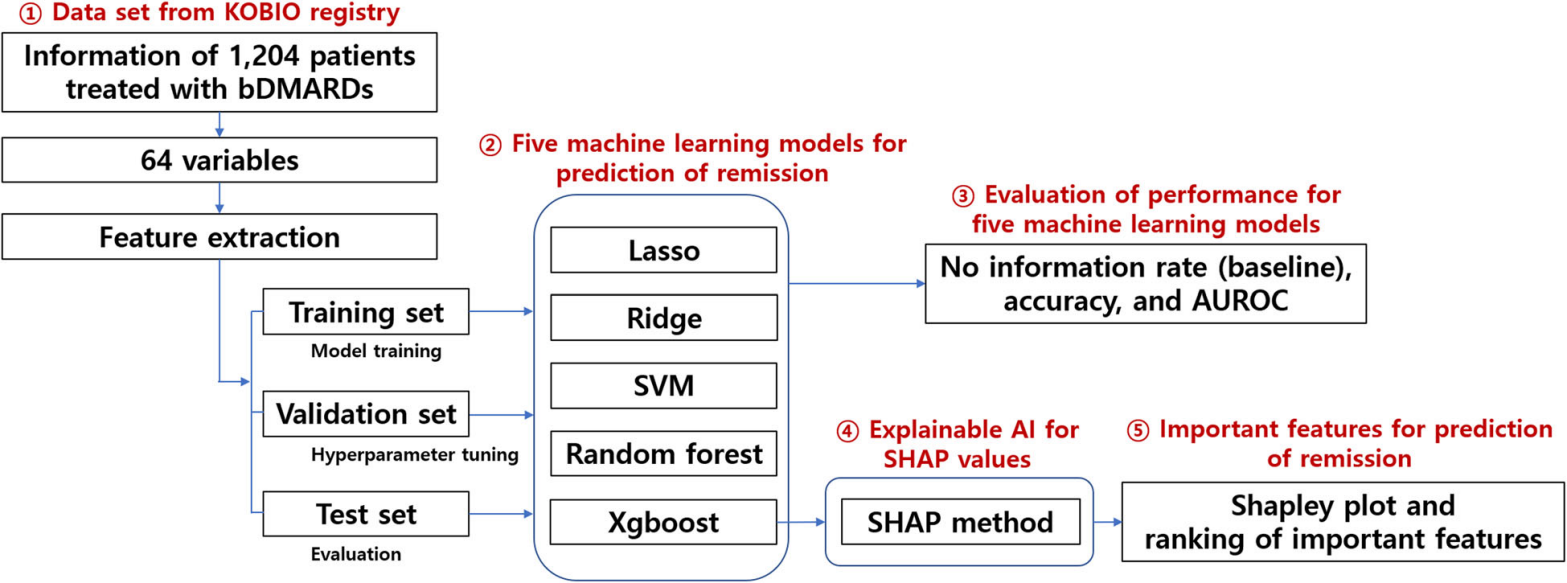
Overview of the study. KOBIO, The Korean College of Rheumatology Biology; bDMARDs, biologic disease-modifying anti-rheumatic drugs; AUROC, area under the receiver operating characteristics; SVM, support vector machine; AI, artificial intelligence

The disease activity scores in 28 joints using the erythrocyte sedimentation rate (DAS28-ESR) were measured at baseline establishment and during follow-up. The outcome of the prediction model evaluating the responses to bDMARDs was “remission” at follow-up, which was defined as DAS28-ESR ≤ 2.6 at follow-up. To prevent overestimation of remission by prednisolone treatment, another prediction model was constructed with the outcome of “remission without increasing prednisolone dose”. An overview of the study flow is presented in Fig. 2.

Five machine learning models were used to predict remission in subjects receiving bDMARDs, tumor necrosis factor (TNF) inhibitors, non-TNF inhibitors, and each bDMARD; the models included lasso and ridge based on linear relationships [14], support vector machine using kernel methods [15], tree-based random forest [16], and Xgboost [17]. To avoid overfitting problems, the training and test sets were divided in a 7:3 ratio, and the models were trained with the training set; then, the prediction results were verified using the test set. For the training dataset, a 5-fold cross validation was performed to tune the hyperparameters determined as outside models (Additional file: Table S1 and Table S2). In this procedure, a grid search was conducted to evaluate all possible combinations of the hyperparameters. The grid search found optimal hyperparameters with the objective function of determining the area under the receiver operating characteristics (AUROC) in each model. Bootstrapping (random sampling with replacement) was also performed to obtain a median value for the AUROC curve and to determine the accuracy for reducing measurement variances caused by small samples when dividing between the training and test sets.

Dimension reduction was performed to avoid the “curse of dimensionality” caused by a large number of variables compared with the size of the data. Among the 64 variables, we selected variables that are frequently encountered in clinical practice for prescription of biologics and excluded variables that are not referenced when prescribing biologics. As a result, 15 variables known to be of clinical importance were preselected (i.e., sex, age, baseline DAS28-ESR, methotrexate dose, steroid dose, erythrocyte sedimentation rate [ESR], C-reactive protein [CRP], rheumatoid factor [RF], anti-cyclic citrullinated peptide antibody [ACPA], anti-nuclear antibody [ANA], and five comorbidities). Subsequently, 20 variables that were highly correlated with the drug response (remission) of each bDMARD were selected. After selecting variables based on data, we created a prediction model by training with a fixed set of 35 variables.

Missing data for variables (Additional file: Table S3) were replaced with the median value for each variable. With a similar logic, binary variables such as comorbidities were coded as 1 if “yes” and 0 if “no” or “no test” because “no” was the most common value.

### XAI for identifying important clinical features associated with responses to bDMARDs

Using the Xgboost machine learning model, the important variables and their impact on predicting remissions using XAI were determined. By focusing on improving the performance and accuracy, machine learning models have become complex, and their interpretability has declined. Although there are some feature importance measures in random forest and Xgboost, these models provide inconsistent results depending on the tree structure; in addition, they only show the overall importance and not the direction of the effect of the independent variables [12]. To overcome these issues, the Shapley additive explanations (SHAP) method was developed [18], which approximates a complex model to a linear model and interprets the feature importance in the linear model to demonstrate the amount by which a given feature changes the prediction. In addition, XAI provides a Shapley plot that can be easily explained visually and easily to understand the complex relationship between variables and outcomes compared to random forest. This methodology satisfies three conditions: (1) the approximated linear model has a similar accuracy to the original model in the local domain, (2) meaningless variables have no impact on the explanatory power of the model, and (3) feature importance is consistent in the model structure. Accordingly, the SHAP method demonstrates consistent feature importance regardless of model structure and direction of effect of the predictive variables, thereby allowing clinicians to acquire insights into achieving remission and to find potential variables affecting the selection of appropriate bDMARDs.

### Statistical analysis

All data are shown as mean (standard deviation [SD]) or percentage values. To evaluate the machine learning performance, the accuracy and AUROC curve were analyzed. The no information rate, which is the largest proportion of the observed classes, was used as a baseline to determine the overall distribution of the classification and to compare with those of the machine learning models. Statistical analyses were performed using R software version 3.6.1 (R Foundation for Statistical Computing, Vienna, Austria), and model training was performed using the caret package and SHAPforxgboost package in R.

## Results

### Clinical characteristics of the patients

Table 1 shows the clinical characteristics of the 1204 patients included in the study. The mean (SD) age at baseline was 54.0 (12.8) years and the majority (82.6%) of the patients were female. The mean (SD) disease duration was 7.1 (7.2) years. The rheumatoid factor (RF) and ACPA positivity were 83.2% and 73.4%, respectively, and the mean (SD) DAS28-ESR values at baseline and follow-up were 5.6 (1.0) and 4.3 (1.3), respectively. The mean (SD) duration from the initiation of biologics to the next visit was 0.97 (0.31) years. Of the 1397 follow-up data, 546 reached remission that is not more than 2.6 of DAS28-ESR; in those follow-up data, the mean (SD) number of follow-up visits and the duration of follow-up until reaching remission was 2.2 times (0.59) and 1.1 years (0.35), respectively.

**Table 1:** Clinical characteristics of the 1204 patients treated with biologics

Variable	Value
Age, mean (SD), year	54.0 (12.8)
Female (%)	82.6
Disease duration, mean (SD), year	7.1 (7.2)
Non-smoking (%)	84.4
History of cardiovascular diseases (%)	3.9
History of lung diseases (%)	6.1
History of hemato-oncologic diseases (%)	1.3
HBsAg positivity (%)	3.5
HBsAb positivity (%)	46.7
HBcAb positivity (%)	7.6
HCV Ab positivity (%)	0.75
Rheumatoid factor positivity (%)	83.2
Rheumatoid factor, mean (SD), mg/dL	141.6 (216.8)
Anti-CCP antibody positivity (%)	73.4
Anti-CCP antibody, mean (SD), mg/dL	190.3 (242.1)
Erythrocyte sedimentation rate, mean (SD), mm^3^/h	48.6 (26.8)
C-reactive protein, mean (SD), mg/dL	2.4 (3.0)
Anti-nuclear antibody (%)	35.4
Methotrexate treatment (%)	83.7
Methotrexate dose, mean (SD), mg	10.5 (5.4)
Treatment of cDMARDs other than methotrexate (%)^a^	33.6
Prednisolone treatment (%)^b^	71.1
Prednisolone dose, mean (SD), mg^b^	5.0 (3.8)
DAS28-ESR at baseline, mean (SD)	5.6 (1.0)
DAS28-ESR at follow-up, mean (SD) (N = 1397)	4.3 (1.3)

^a^Including leflunomide, sulfasalazine, hydroxychloroquine, or tacrolimus

^b^Glucocorticoid dose (e.g., prednisolone, methylprednisolone, deflazacort, and dexamethasone) was converted to prednisolone doses

*cDMARDs* conventional disease-modifying anti-rheumatic drugs, *DAS28-ESR* disease activity score in 28 joints using erythrocyte sedimentation rate, *HBsAg* hepatitis B surface antigen, *HBsAb* hepatitis B surface antibody, *HBcAb* hepatitis B core antibody, *HCV* hepatitis C virus

### Prediction of remission from the five machine learning methods

In all machine learning methods for predicting remission, the accuracy and AUROC curve values were in the ranges of 52.8–72.9% and 0.512–0.694, respectively (Table 2). The ranges of the accuracy and AUROC curve for remission prediction were 59.8–62.0% and 0.596–0.619 in all bDMARDs, 68.4–70.0% and 0.633–0.655 in TNF inhibitors, and 52.8–58.3% and 0.538–0.607 in non-TNF inhibitors, respectively. Among the bDMARDs, the ranges for accuracy and AUROC curve were 67.4–69.8% and 0.623–0.688 for adalimumab, 64.6–67.7% and 0.619–0.656 for etanercept, 61.1–66.7% and 0.615–0.694 for golimumab, 64.6–72.9% and 0.511–0.626 for infliximab, 63.2–68.4% and 0.598–0.679 for abatacept, and 56.1–61.0% and 0.512–0.556 for tocilizumab, respectively. For each bDMARD, the accuracy and AUROC curve were similar across the different machine learning models.

**Table 2:** Prediction of remission for bDMARDs

	Follow-up period (year), mean (sd)	Remission/total	Measurement	Baseline	Lasso	Ridge	SVM	Random forest	Xgboost
**All bDMARDs**	0.96 (0.30)	564/1397 (40.4%)	**Sensitivity**	0.0%	33.1%	29.6%	24.9%	0.6%	32.2%
			**Specificity**	100.0%	79.9%	83.1%	85.1%	100.0%	80.5%
			**Accuracy**	59.6%	61.0%	61.5%	60.5%	59.8%	62.0%
			**AUROC**	0.500	0.614	0.619	0.602	0.608	0.596
**TNF inhibitors**	0.93 (0.32)	252/793 (31.8%)	**Sensitivity**	0.0%	10.7%	21.3%	9.3%	0.0%	20.0%
			**Specificity**	100.0%	97.5%	92.6%	96.0%	100.0%	91.4%
			**Accuracy**	68.2%	69.6%	70.0%	68.4%	68.4%	68.8%
			**AUROC**	0.500	0.649	0.655	0.633	0.644	0.637
**Non-TNF inhibitors**	1.01 (0.27)	312/604 (51.7%)	**Sensitivity**	0.0%	62.4%	64.5%	62.4%	75.3%	57.0%
			**Specificity**	100.0%	52.3%	51.7%	51.7%	33.3%	48.9%
			**Accuracy**	51.7%	57.8%	57.8%	58.3%	55.3%	52.8%
			**AUROC**	0.500	0.605	0.607	0.606	0.586	0.538
**Adalimumab**	0.93 (0.31)	91/289 (31.5%)	**Sensitivity**	0.0%	22.2%	29.6%	37.0%	0.0%	14.8%
			**Specificity**	100.0%	91.5%	88.1%	81.4%	100.0%	93.2%
			**Accuracy**	68.5%	69.8%	69.8%	67.4%	68.6%	69.8%
			**AUROC**	0.500	0.680	0.688	0.663	0.623	0.629
**Etanercept**	0.99 (0.34)	75/220 (34.1%)	**Sensitivity**	0.0%	29.5%	36.4%	40.9%	0.0%	22.7%
			**Specificity**	100.0%	86.0%	83.7%	79.1%	100.0%	88.4%
			**Accuracy**	65.9%	67.7%	67.7%	66.2%	66.2%	64.6%
			**AUROC**	0.500	0.634	0.656	0.643	0.656	0.619
**Golimumab**	0.97 (0.30)	41/122 (33.6%)	**Sensitivity**	0.0%	0.0%	41.7%	50.0%	0.0%	16.7%
			**Specificity**	100.0%	100.0%	79.2%	66.7%	100.0%	95.8%
			**Accuracy**	66.4%	66.7%	63.9%	61.1%	66.7%	66.7%
			**AUROC**	0.500	0.615	0.694	0.623	0.659	0.635
**Infliximab**	0.85 (0.32)	45/162 (27.8%)	**Sensitivity**	0.0%	30.8%	23.1%	30.8%	0.0%	15.4%
			**Specificity**	100.0%	82.9%	88.6%	80.0%	100.0%	85.7%
			**Accuracy**	72.2%	66.7%	70.8%	64.6%	72.9%	66.7%
			**AUROC**	0.500	0.595	0.626	0.544	0.600	0.511
**Abatacept**	0.99 (0.30)	62/194 (32.0%)	**Sensitivity**	0.0%	11.1%	30.6%	38.9%	0.0%	27.8%
			**Specificity**	100.0%	94.9%	84.6%	75.6%	100.0%	82.1%
			**Accuracy**	68.0%	68.4%	68.4%	63.2%	68.4%	64.9%
			**AUROC**	0.500	0.635	0.679	0.636	0.618	0.598
**Tocilizumab**	1.01 (0.26)	250/410 (61.0%)	**Sensitivity**	0.0%	76.0%	81.3%	80.0%	80.0%	77.3%
			**Specificity**	100.0%	29.2%	22.9%	22.9%	21.9%	22.9%
			**Accuracy**	61.0%	57.7%	58.5%	61.0%	56.9%	56.1%
			**AUROC**	0.500	0.552	0.555	0.556	0.522	0.512

*bDMARDs* adalimumab, etanercept, golimumab, infliximab, abatacept, and tocilizumab, *TNF inhibitors* adalimumab, etanercept, golimumab, infliximab, *non-TNF inhibitors* abatacept and tocilizumab. *N* total number of samples for the drug category. Baseline accuracy: remission rate not achieved by clinicians except for tocilizumab and non-TNF inhibitors; baseline AUROC: the value when selecting random or one side

### Important features for remission in XAI

**Fig. 3:**
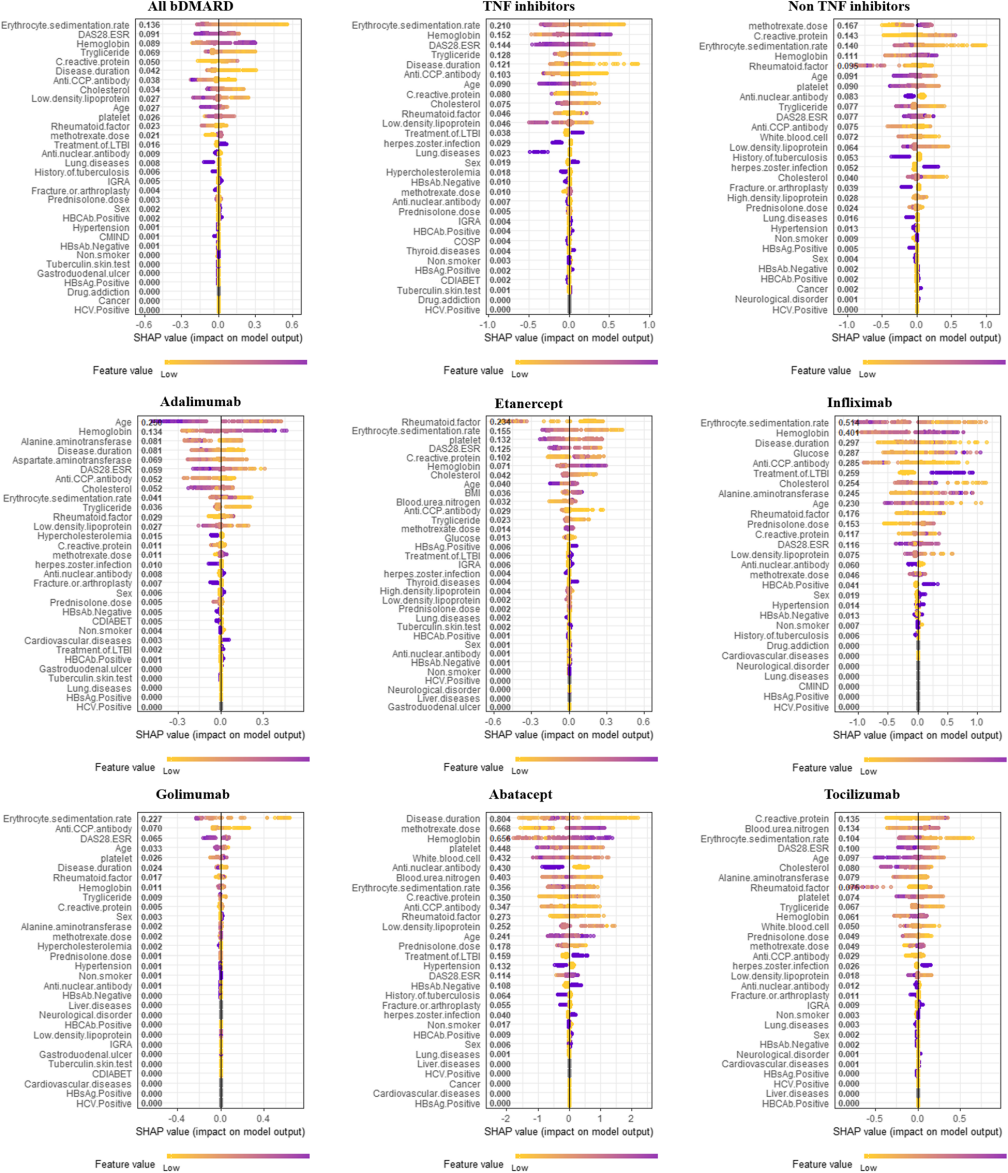
Shapley plots for bDMARDs. Shapley plots show the SHAP values in the order of the important variables that contribute to remission. The feature values change from light (low) to dark (high) for each variable. For age, for example, the lighter colors represent lower age, whereas the darker colors represent higher age. For adalimumab, increasing (positive) SHAP values with lighter colors indicate that lower ages are strongly related to remission, whereas decreasing (negative) SHAP values with darker colors indicate that higher ages are strongly related to remission failure. Therefore, linear changes in the feature values are expressed in color, and the SHAP values (impact on model output) indicate closer to remission or remission failure based on the feature values. DAS, disease activity score; ESR, erythrocyte sedimentation rate; LTBI, latent tuberculosis infection; IGRA, interferon-gamma release assay; HBcAb, hepatitis B core antibody; CMIND, psychiatric comorbidity; HBsAb, hepatitis B surface antibody; HBsAg, hepatitis B surface antigen; HCV, hepatitis C virus; COSP, osteoporosis; CDIABET, diabetes mellitus and its complications; BMI, body mass index

The SHAP method for remission was used to determine the influences of the variables that contributed to remission in the prediction model. The interpretations of the feature importance with the Shapley plot are shown in Fig. 3 and Additional file: Table S4, where the features are listed in order of their absolute values.

In all bDMARDs, the ESR was the most important feature for predicting remission (SHAP value = − 0.136), with low and high ESR levels associated with remission and remission failure, respectively. The DAS28-ESR was the second most important feature and had a negative association with remission (− 0.091). The hemoglobin was the third most important feature and had a positive association with remission (0.089). In TNF inhibitors, ESR was the most important feature (− 0.210), followed by hemoglobin and DAS28-ESR (0.152 and − 0.144, respectively). In non-TNF inhibitors, methotrexate dose was the most important feature (0.167), followed by CRP and ESR (0.143 and − 0.140, respectively).

Age was the most important feature for adalimumab (− 0.250), fourth for golimumab (− 0.033), and fifth for tocilizumab (− 0.097). RF was the most important feature for etanercept (− 0.234), but it was not among the top few features for the other bDMARDs. ESR was the most important feature for infliximab (− 0.514) and golimumab (− 0.227) and was the most important feature of most bDMARDs. Disease duration was the most important feature for abatacept (− 0.804), third for infliximab (− 0.297), and sixth for golimumab (− 0.024). CRP was the most important feature for tocilizumab (0.135) and fifth important feature for etanercept (0.102); however, CRP was not among the top features for the other bDMARDs.

### Rankings of important features in bDMARDs

Table 3 shows the order of the average of the SHAP value ranks from the Shapley plot in the bDMARDs. The degree and direction of contribution of the variables to remission were different for each bDMARD. Among the variables, the ESR had the highest average ranking (4.0) and had a negative association with remission for all bDMARDs. Hemoglobin had the second-highest average ranking (5.3) and had a positive association with remission for all bDMARDs except for golimumab (no association). The age and DAS28-ESR factors had the third and fourth highest average rankings (6.67 and 7.83, respectively) and had negative associations with remissions for adalimumab, etanercept, infliximab, golimumab, and tocilizumab. For abatacept, DAS28-ESR was positively associated with remission, and age had a nonlinear relationship with remission.

**Table 3:** Rankings of the important features for the prediction of remission in each biologic

Clinical feature	Average ranking	Adalimumab	Etanercept	Infliximab	Golimumab	Abatacept	Tocilizumab
ESR	4.0	− 0.041	− 0.155	− 0.514	− 0.227	− 0.356	− 0.104
Hemoglobin	5.3	+ 0.134	+ 0.071	+ 0.401	0	+ 0.656	+ 0.061
Age	6.7	− 0.250	− 0.040	− 0.230	− 0.033	^a^0.241	− 0.097
DAS28-ESR	7.8	− 0.059	− 0.125	− 0.116	− 0.065	+ 0.114	− 0.100
Rheumatoid factor	8.0	+ 0.029	− 0.234	^a^0.176	0	0.273^a^	− 0.075
Anti-CCP antibody	8.3	− 0.052	− 0.029	− 0.285	− 0.070	− 0.347	+ 0.029
CRP	8.7	0	+ 0.102	+ 0.117	0	+ 0.350	+ 0.135
Disease duration	11.5	− 0.081	0	0.297^a^	− 0.024	− 0.804	0
Methotrexate dose	11.7	0	0	0.046^a^	0	+ 0.668	+ 0.049
Platelet	11.8	0	− 0.132	0	− 0.026	− 0.448	− 0.074
Cholesterol	12.5	− 0.052	− 0.042	− 0.254	0	0	− 0.080
ALT	15.0	− 0.081	0	+ 0.245	0	0	− 0.079
BUN	15.0	0	+ 0.032	0	0	− 0.403	+ 0.134
Triglyceride	16.0	− 0.036	− 0.023	0	0	0	− 0.067
ANA	16.0	0	0	− 0.060	0	− 0.430	0

^a^A nonlinear relationship such as quadratic effect or mixed effect between drugs and variables. The average ranking was obtained by averaging the rankings of the 6 bDMARDs

*ESR* erythrocyte sedimentation rate, *DAS28-ESR* disease activity scores in 28 joints using the erythrocyte sedimentation rate, *CRP* C-reactive protein, *ALT* alanine aminotransferase, *BUN* blood urea nitrogen, *ANA* anti-nuclear antibody

The relationship between RF levels and remission was different for each bDMARD. RF was positively associated with remission in adalimumab, but negatively associated with remission in etanercept and tocilizumab. RF had a nonlinear relationship with remission in infliximab and abatacept, and no association with remission in golimumab. In terms of ACPA, low ACPA was associated with remission in all bDMARDs (negative association) except tocilizumab (positive association). The elevation of CRP was associated with remission in bDMARDs (positive association) except for adalimumab and golimumab (no association). In most bDMARDs, disease duration, platelet, cholesterol, and triglyceride showed negative associations with remission. ANA had a negative association with remission for infliximab and abatacept.

### Predictive model for remission without increasing prednisolone dose

We built a machine learning model for remission without increasing prednisolone dose as another outcome (Additional file: Table S5). Among the 1397 follow-ups, 537 were classified as remission without increasing prednisolone dose. The ranges of accuracy and AUROC were 54.1–72.9% and 0.517–0.698, respectively, which were similar to the results for predicting remission. In the Shapley plots, compared with the outcomes for predicting remission, the SHAP scores had slight fluctuations while the order of the variables was similar (Additional file: Figure S1 and Table S6). In the ranking of important features, there was a slight change due to the difference in the SHAP value of each variable (Additional file: Table S7). However, important features such as ESR, DAS28-ESR, CRP, age, hemoglobin, RF, and ACPA retained high rankings for predicting remission.

## Discussion

In the prediction models of remission with all machine learning methods, the ranges of accuracy and AUROC were 52.8–72.9% and 0.512–0.694, respectively. Notably, based on the machine learning models for predicting remission, we identified important clinical features that were associated with remission after treatment with bDMARDs. Although it is possible to estimate the importance of each variable by linear regression analysis, we determined that machine learning would complement statistical methods due to the complex relationships between the variables and remission. Some significant clinical features for remission prediction were commonly identified in the bDMARDs, albeit with slight differences in the impact between the bDMARDs.

Guan et al. used machine learning to predict the responses to TNF inhibitors in patients with RA using clinical and genetic markers [10]; the authors created a Gaussian process regression model to predict the changes in DAS28 and classified the patients into responders and non-responders. However, Guan et al. used variables that are difficult to obtain in routine practice such as genetic variables, and their outcomes were different from our study. Norgeot et al. used a longitudinal deep learning model to predict controlled (remission or low activity) or uncontrolled state (moderate or high activity) with clinical disease activity index in the next clinical visit [11]; their study showed good performance, but it was not aimed at predicting the therapeutic response of the biologics, but rather predicting the clinical disease activity index at the next visit. Because the goal of our study was to predict the response of biologics, it is difficult to compare our prediction of remission with their outcome.

As opposed to how machine learning models, such as those using deep learning are “black-box” models to explain the reason for prediction, the XAI machine learning method provides reasons for prediction in a manner that lay users can easily understand. For interpretability, the SHAP method, which is based on the Shapley values, was recently presented [18]. The Shapley value employs a method based on game theory, which was introduced to suggest how to fairly distribute payout among the features [19]. This theory has influenced various fields and has recently been used in machine learning to improve the interpretability of complex models. The SHAP method is a unified framework that improves interpretability while maintaining predictability of complex models with machine learning. Using the SHAP method, we identified the characteristics of the important variables that contribute to prediction of remission in a complex cohort dataset. In the use of healthcare data, it is important to discern the relationships between the variables in clinical, genomic, and other types of healthcare data. This proposed method is expected to provide insights for finding the relationships among numerous variables in the integration of large-scale healthcare data.

Among various baseline variables, several laboratory findings were identified as important features that ranked highly for predicting remission. For all the bDMARDs, the most important feature was the ESR, which is a variable predictive of disease activity [20, 21]. Hemoglobin was the second most important feature in our study; considering how anemia is associated with disease activity [22] and erosion progression [23], hemoglobin may be an important factor indicating disease states in patients with RA. The RF and ACPA were also important for prediction of remission and contributed to determination of the direction of treatment in patients with RA [24, 25]. However, several studies have shown conflicting results on the relationship between RF and ACPA in response to TNF inhibitors [26, 27]. In our study, the RF and ACPA were associated with remission in various degrees of impact and direction for each bDMARD. Moreover, RF was not linearly related to remission for infliximab and abatacept; as such, changes in the feature value were not significantly related to either remission or remission failure.

This study has some obvious limitations. First, the dosage intervals and doses of biologics were not considered. Second, this study did not distinguish between the primary response failure (i.e., failure of clinical improvement) and secondary response failure (i.e., loss of response after clinical improvement) [27]. Third, this study did not provide evidence as to how the important features of each bDMARD were related to their mechanisms of action. Fourth, because this study focused on the important features of the variables needed for prediction in the machine learning model, the performance of machine learning was not confirmed using external test sets from other RA cohort data. Fifth, because this study included patients treated with first bDMARDs and excluded those who had been previously treated with bDMARDs, such patient characteristics should be considered in the interpretation of the Shapley plot. Sixth, the prediction of response to biologics is difficult because it can be affected by individual variances. In addition, it is difficult to enhance the predictive power because there is a limit in the amount of variables that can be obtained in routine clinical practice. Importantly, the problem of missing values in real-world data is one of the limitations of this study. Lastly, although the KOBIO registry is an inception cohort of consecutive patients treated with biologics, those who failed to meet the inclusion criteria or did not consent to participate in the registry were not included, which may lead to a selection bias.

## Conclusion

We successfully developed machine learning models to predict remission as a response to different bDMARDs in active RA patients based on their clinical profiles. Using these models, the XAI was able to identify important clinical features associated with remission according to the biologics used. We noted that some important features were more strongly associated with remission, albeit the order of their relative importance was different for each biologic. Our results suggest that an advanced machine learning approach may be helpful for supporting clinical decisions to improve treatment outcomes with biologics in RA patients.

### Additional contributions

The registry was funded by the Korean College of Rheumatology, which had no involvement in the study design, collection, analysis, and interpretation of the data; in writing the manuscript; or in the decision to submit the manuscript for publication. We would like to thank all members of The Korean College of Rheumatology Biology (KOBIO) registry. We thank Dr. Joon Seo Lim from the Scientific Publications Team at Asan Medical Center for his editorial assistance with preparing this manuscript.

## Supplementary Information

**Additional file 1: Table S1**. Hyperparameters for bDMARDs. **Table S2**. Hyperparameters for prediction of remission without increasing prednisolone dose. **Table S3**. Proportions of missing data in each variable. **Table S4**. SHAP values for the variables in Shapely plots. **Table S5**. Prediction of remission without increasing prednisolone dose for bDMARD. **Table S6**. SHAP values for variables in Shapely plots. **Table S7**. Order of important features for prediction of remission without increasing prednisolone dose in each bDMARD. **Figure S1**. Shapley plots predicted.

## Data Availability

Data are available from the Clinical Research Committee of KOBIO under the Korean College of Rheumatology for researchers who meet the criteria for access to confidential data. To request data, please contact Kichul Shin, MD, PhD, Director of the Korean College of Rheumatology Biologics Registry, Associate Professor of the Division of Rheumatology, Director of Logistics Planning at SMG-SNU, Boramae Medical Center, 20 Boramae-ro-5-gil, Dongjak-gu, Seoul, 07061, Korea; Tel: 822 870 3204; Fax: 822 870 3866; Email: rk.ca.uns@1bedik.

## Abbreviations

ACPA: Anti-cyclic citrullinated peptide antibody
ANA: Anti-nuclear antibody
AUROC: Area under the receiver operating characteristics
bDMARDs: Biologic disease-modifying anti-rheumatic drugs
cDMARDs: Conventional disease-modifying anti-rheumatic drugs
CRP: C-reactive protein
ESR: Erythrocyte sedimentation rate
DAS28-ESR: Disease activity scores in 28 joints using the erythrocyte sedimentation rate
KOBIO: Korean College of Rheumatology Biologics and Targeted Therapy Registry
RA: Rheumatoid arthritis
RF: Rheumatoid factor
TNF: Tumor necrosis factor
SHAP: Shapley additive explanations
SD: Standard deviation
tsDMARDs: Targeted synthetic disease-modifying anti-rheumatic drugs
XAI: Explainable artificial intelligence
