# Supplementary material for: Machine learning model for identifying important clinical features for predicting remission in patients with rheumatoid arthritis treated with biologics
Source: Arthritis Res Ther. 2021 Jul 6;23:178. doi: 10.1186/s13075-021-02567-y (PMC8259419; doi:10.1186/s13075-021-02567-y)
Supplement: Supplementary file 1 — Additional file 1: Table S1. Hyperparameters for bDMARDs. Table S2. Hyperparameters for prediction of remission without increasing prednisolone dose. Table S3. Proportions of missing data in each variable. Table S4. SHAP values for the variables in Shapely plots. Table S5. Prediction of remission without increasing prednisolone dose for bDMARD. Table S6. SHAP values for variables in Shapely plots. Table S7. Order of important features for prediction of remission without increasing prednisolone dose in each bDMARD. Figure S1. Shapley plots predicted. [file 13075_2021_2567_MOESM1_ESM.docx]

**SUPPLEMENTARY RESULTS**

**Table S1. Hyperparameters for bDMARDs.**

|  | **Lasso, lambda** | **Ridge, lambda** | **SVM, cost** | **Random forest mtry** | **XGB, nrounds** | **XGB, depth** | **XGB, eta** | **XGB, gamma** |
| --- | --- | --- | --- | --- | --- | --- | --- | --- |
| **All bDMARDs** | 0 | 0.05 | 150 | 1 | 1900 | 5 | 0.2 | 6 |
| **TNF inhibitors** | 0.02 | 0.05 | 50 | 1 | 100 | 5 | 0.2 | 6 |
| **Non-TNF inhibitors** | 0 | 0.046 | 50 | 1 | 1800 | 5 | 0.3 | 6 |
| **Adalimumab** | 0.02 | 0.047 | 10 | 1 | 300 | 35 | 0.2 | 6 |
| **Etanercept** | 0.02 | 0.035 | 10 | 1 | 900 | 50 | 0.3 | 6 |
| **Golimumab** | 0.16 | 0.033 | 10 | 1 | 100 | 65 | 0.2 | 6 |
| **Infliximab** | 0 | 0.049 | 70 | 1 | 100 | 35 | 0.1 | 0 |
| **Abatacept** | 0.04 | 0.05 | 150 | 1 | 600 | 35 | 0.3 | 0 |
| **Tocilizumab** | 0 | 0.046 | 190 | 13 | 100 | 65 | 0.3 | 6 |

**Table S2. Hyperparameters for prediction of remission without increasing prednisolone dose.**

|  | **Lasso, lambda** | **Ridge, lambda** | **SVM, cost** | **Random forest mtry** | **XGB, nrounds** | **XGB, depth** | **XGB, eta** | **XGB, gamma** |
| --- | --- | --- | --- | --- | --- | --- | --- | --- |
| **All bDMARDs** | 0 | 0.05 | 70 | 3 | 300 | 20 | 0.1 | 12 |
| **TNF inhibitors** | 0 | 0.05 | 90 | 1 | 1300 | 50 | 0.1 | 6 |
| **Non-TNF inhibitors** | 0 | 0.035 | 130 | 1 | 1400 | 5 | 0.3 | 6 |
| **Adalimumab** | 0.02 | 0.05 | 30 | 9 | 900 | 35 | 0.1 | 6 |
| **Etanercept** | 0 | 0.038 | 90 | 1 | 1900 | 20 | 0.2 | 6 |
| **Golimumab** | 0.14 | 0.046 | 70 | 25 | 2000 | 20 | 0.1 | 6 |
| **Infliximab** | 0 | 0.049 | 70 | 1 | 100 | 35 | 0.1 | 0 |
| **Abatacept** | 0.02 | 0.046 | 50 | 1 | 1300 | 35 | 0.2 | 6 |
| **Tocilizumab** | 0 | 0.05 | 10 | 5 | 1900 | 50 | 0.3 | 6 |

**Table S3. Proportions of missing data in each variable.**

| **Variable** | **%** |
| --- | --- |
| History of tuberculosis | 74.74 |
| Tuberculin skin test | 90.16 |
| IGRA | 76.87 |
| History of LTBI | 65.62 |
| HBs antigen | 78.80 |
| HBs antibody | 82.91 |
| HBc antibody | 94.40 |
| Hepatitis C virus infection | 85.35 |
| History of herpes zoster infection | 75.91 |
| ANA | 68.44 |
| RF | 8.88 |
| ACPA | 30.98 |
| Alkaline phosphatase | 17.97 |
| BUN | 9.56 |
| Creatinine | 7.95 |
| Cholesterol | 26.15 |
| HDL | 63.99 |
| LDL | 69.80 |
| TG | 58.56 |
| Glucose | 20.49 |

ACPA, anti-CCP antibody; ANA, anti-nuclear antibody; HBsAg, Hepatitis B surface antigen; HBsAb, Hepatitis B surface antibody; HBcAb, Hepatitis B core antibody; BUN, blood urea nitrogen; HDL, high-density lipoprotein; IGRA, interferon-gamma release assay; LDL, low-density lipoprotein; LTBI, latent tuberculosis infection; RF, rheumatoid factor; TG; triglyceride.

**Table S4. SHAP values for the variables in Shapely plots.**

| **Ranking** | **All bDMARDs** | **TNF inhibitor** | **Non-TNF inhibitor** | **Adalimumab** | **Etanercept** | **Infliximab** | **Golimumab** | **Abatacept** | **Tocilizumab** |
| --- | --- | --- | --- | --- | --- | --- | --- | --- | --- |
| 1 | ESR  0.136 | ESR  0.210 | MTX dose  0.167 | Age  0.256 | RF  0.234 | ESR  0.514 | ESR  0.227 | Disease duration  0.804 | CRP  0.135 |
| 2 | DAS28-ESR  0.091 | Hemoglobin  0.152 | CRP  0.143 | Hemoglobin  0.134 | ESR  0.155 | Hemoglobin  0.401 | ACPA  0.070 | MTX dose  0.668 | BUN  0.134 |
| 3 | Hemoglobin  0.089 | DAS28-ESR  0.144 | ESR  0.140 | ALT  0.081 | Platelet  0.132 | Disease duration  0.297 | DAS28-ESR  0.065 | Hemoglobin  0.656 | ESR  0.104 |
| 4 | TG  0.069 | TG  0.128 | Hemoglobin  0.111 | Disease duration  0.081 | DAS28-ESR  0.125 | Glucose  0.287 | Age  0.033 | Platelet  0.448 | DAS28-ESR  0.100 |
| 5 | CRP  0.050 | Disease duration  0.121 | RF  0.095 | AST  0.069 | CRP  0.102 | ACPA  0.285 | Platelet  0.026 | White blood cell  0.432 | Age  0.097 |
| 6 | Disease duration  0.042 | ACPA  0.103 | Age  0.091 | DAS28-ESR  0.059 | Hemoglobin  0.071 | Treatment of LTBI  0.259 | Disease duration  0.024 | ANA  0.430 | Cholesterol  0.080 |
| 7 | ACPA  0.038 | Age  0.090 | Platelet  0.090 | ACPA  0.052 | Cholesterol  0.042 | Cholesterol  0.254 | RF  0.017 | BUN  0.403 | ALT  0.079 |
| 8 | Cholesterol  0.034 | CRP  0.080 | ANA  0.083 | Cholesterol  0.052 | Age  0.040 | ALT  0.245 | Hemoglobin  0.011 | ESR  0.356 | RF  0.076 |
| 9 | LDL  0.027 | Cholesterol  0.075 | TG  0.077 | ESR  0.041 | BMI  0.036 | Age  0.230 | TG  0.009 | CRP  0.350 | Platelet  0.074 |
| 10 | Age  0.027 | RF  0.046 | DAS28-ESR  0.077 | TG  0.036 | BUN  0.032 | RF  0.176 | CRP  0.005 | ACPA  0.347 | TG  0.067 |
| 11 | Platelet  0.026 | LDL  0.046 | ACPA  0.075 | RF  0.029 | ACPA  0.029 | Prednisolone dose  0.153 | Sex  0.003 | RF  0.273 | Hemoglobin  0.061 |
| 12 | RF  0.023 | Treatment of LTBI  0.038 | White blood cell  0.072 | LDL  0.027 | TG  0.023 | CRP  0.117 | ALT  0.002 | LDL  0.252 | White blood cell  0.050 |
| 13 | MTX dose  0.021 | History of herpes zoster  0.029 | LDL  0.064 | Hypercholesterolemia  0.015 | MTX dose  0.014 | DAS28-ESR  0.116 | MTX dose  0.002 | Age  0.241 | Prednisolone dose  0.049 |
| 14 | Treatment of LTBI  0.016 | Lung disease  0.023 | History of tuberculosis  0.053 | CRP  0.011 | Glucose  0.013 | LDL  0.075 | Hypercholesterolemia  0.002 | Prednisolone dose  0.178 | MTX dose  0.049 |
| 15 | ANA  0.009 | Sex  0.019 | History of herpes zoster  0.052 | MTX dose  0.011 | HBs antigen positive  0.006 | ANA  0.060 | Prednisolone dose  0.001 | Treatment of LTBI  0.159 | ACPA  0.029 |
| 16 | Lung disease  0.008 | Hypercholesterolemia  0.018 | Cholesterol  0.040 | History of herpes zoster  0.010 | Treatment of LTBI  0.006 | MTX dose  0.046 | Hypertension  0.001 | Hypertension  0.132 | History of herpes zoster  0.026 |
| 17 | History of tuberculosis  0.006 | HBs antibody negative  0.010 | Fracture of arthroplasty  0.039 | ANA  0.008 | IGRA  0.006 | HBC antibody positive  0.041 | Non-smoker  0.001 | DAS28-ESR  0.114 | LDL  0.018 |
| 18 | IGRA  0.005 | MTX dose  0.010 | HDL | Fracture or arthroplasty  0.007 | History of herpes zoster  0.004 | Sex  0.019 | ANA  0.001 | HBs antibody negative  0.108 | ANA  0.012 |
| 19 | Fracture or arthroplasty  0.004 | ANA  0.007 | Prednisolone dose  0.024 | Sex  0.006 | Thyroid diseases  0.004 | Hypertension  0.014 | HBs antibody negative  <0.001 | History of tuberculosis  0.064 | Fracture of arthroplasty  0.011 |
| 20 | Prednisolone dose  0.003 | Prednisolone dose  0.005 | Lung diseases  0.016 | Prednisolone dose  0.005 | HDL  0.004 | HBs antibody negative  0.013 | Liver diseases  <0.001 | Fracture or arthroplasty  0.055 | IGRA  0.009 |
| 21 | Sex  0.002 | IGRA  0.004 | Hypertension  0.013 | HBs antibody negative  0.005 | LDL  0.002 | Non-smoker  0.007 | Neurological disorder  <0.001 | History of herpes zoster  0.040 | Non-smoker  0.003 |
| 22 | HBC Antibody positive  0.002 | HBC antibody positive  0.004 | Non-smoker  0.009 | Diabetes mellitus  0.005 | Prednisolone dose  0.002 | History of tuberculosis  0.006 | HBC antibody positive  <0.001 | Non-smoker  0.017 | Lung diseases  0.003 |
| 23 | Hypertension  0.001 | Osteoporosis  0.004 | HBs antigen positive  0.005 | Non-smoker  0.004 | Lung diseases  0.002 | Drug addiction  <0.001 | LDL  <0.001 | HBC antibody positive  0.009 | Sex  0.002 |
| 24 | Psychiatric problem  0.001 | Thyroid diseases  0.004 | Sex  0.004 | Cardiovascular diseases  0.003 | Tuberculin skin test  0.002 | Cardiovascular diseases  <0.001 | IGRA  <0.001 | Sex  0.006 | HBs antibody negative  0.002 |
| 25 | HBs antibody negative  0.001 | Non-smoker  0.003 | HBs antibody negative  0.002 | Treatment of LTBI  0.002 | HBC antibody positive  0.001 | Neurological disorder  <0.001r | Gastroduodenal ulcer  <0.001 | Lung diseases  0.001 | Neurological disorder  0.001 |
| 26 | Non-smoker  <0.001 | HBs antigen positive  0.002 | HBC antibody positive  0.002 | HBC antibody positive  0.001 | Sex  0.001 | Lung diseases  <0.001 | Tuberculin skin test  <0.001 | Liver diseases  <0.001 | Cardiovascular diseases  0.001 |
| 27 | Tuberculin skin test  <0.001 | Diabetes mellitus  0.002 | History of cancer  0.002 | Gastroduodenal ulcer  <0.001 | ANA  0.001 | Psychiatric problem  <0.001 | Diabetes mellitus  <0.001 | HCV positive  <0.001 | HBs antigen positive  <0.001 |
| 28 | Gastroduodenal ulcer  <0.001 | Tuberculin skin test  0.001 | Neurological disorder  0.001 | Tuberculin skin test positive  <0.001 | HBs antibody negative  0.001 | HBs antigen positive  <0.001 | Cardiovascular diseases  <0.001 | History of cancer  <0.001 | HCV positive  <0.001 |
| 29 | HBs antigen positive  <0.001 | Drug addiction  <0.001 | HCV positive  <0.001 | Lung diseases  <0.001 | Non-smoker  <0.001 | HCV positive  <0.001 | HBs antigen positive  <0.001 | Cardiovascular diseases  <0.001 | Liver diseases  <0.001 |
| 30 | Drug addition  <0.001 | HCV positive  <0.001 |  | HBs antigen positive  <0.001 | Neurological disorder  <0.001 |  | HCV positive  <0.001 | HBs antigen positive  <0.001 | HBC antibody positive  <0.001 |
| 31 | History of cancer  <0.001 |  |  | HCV positive  <0.001 | Liver diseases  <0.001 |  |  |  |  |
| 32 | HCV positive  <0.001 |  |  |  | Gastroduodenal ulcer  <0.001 |  |  |  |  |

ACPA, anti-CCP antibody; ALT, alanine aminotransferase; ANA, anti-nuclear antibody; AST, aspartate aminotransferase; HBsAg, Hepatitis B surface antigen; HBsAb, Hepatitis B surface antibody; HBcAb, Hepatitis B core antibody; HCV, hepatitis C virus; BUN, blood urea nitrogen; CRP, C-reactive protein; DAS28-ESR, disease activity scores in 28 joints using the erythrocyte sedimentation rate; ESR, erythrocyte sedimentation rate; HDL, high-density lipoprotein; IGRA, interferon-gamma release assay; LDL, low-density lipoprotein; LTBI, latent tuberculosis infection; MTX, methotrexate; RF, rheumatoid factor; TG; triglyceride.

**Table S5. Prediction of remission without increasing prednisolone dose for bDMARD.**

|  | **Follow-up period (year), mean (sd)** | **Remission / total** | **Measure** | **Baseline** | **Lasso** | **Ridge** | **SVM** | **Random Forest** | **Xgboost** |
| --- | --- | --- | --- | --- | --- | --- | --- | --- | --- |
| **All bDMARDs** | 0.96 (0.30) | 537/1,397  (38.4%) | **Sensitivity** | 0.0% | 28.0% | 23.6% | 9.3% | 28.0% | 10.2% |
|  |  |  | **Specificity** | 100.0% | 84.5% | 88.0% | 94.8% | 84.5% | 95.7% |
|  |  |  | **Accuracy** | 61.6% | 63.2% | 63.6% | 64.4% | 63.4% | 62.3% |
|  |  |  | **AUROC** | 0.500 | 0.610 | 0.616 | 0.617 | 0.619 | 0.604 |
| **TNF inhibitors** | 0.93 (0.32) | 247/793  (31.1%) | **Sensitivity** | 0.0% | 24.3% | 20.3% | 6.1% | 0.0% | 21.6% |
|  |  |  | **Specificity** | 100.0% | 90.2% | 93.9% | 97.9% | 100.0% | 89.6% |
|  |  |  | **Accuracy** | 68.9% | 69.6% | 70.5% | 68.8% | 68.8% | 70.0% |
|  |  |  | **AUROC** | 0.500 | 0.649 | 0.654 | 0.627 | 0.646 | 0.642 |
| **Non-TNF inhibitors** | 1.01 (0.27) | 290/604  (48.0%) | **Sensitivity** | 0.0% | 54.0% | 54.0% | 55.2% | 43.7% | 47.1% |
|  |  |  | **Specificity** | 100.0% | 59.6% | 60.1% | 60.6% | 70.2% | 57.4% |
|  |  |  | **Accuracy** | 52.0% | 56.9% | 56.9% | 57.5% | 56.9% | 54.1% |
|  |  |  | **AUROC** | 0.500 | 0.601 | 0.604 | 0.601 | 0.592 | 0.554 |
| **Adalimumab** | 0.93 (0.31) | 89/289  (30.8%) | **Sensitivity** | 0.0 | 23.1% | 26.9% | 38.5% | 19.2% | 11.5% |
|  |  |  | **Specificity** | 100.0 | 90.0% | 86.7% | 80.0% | 90.0% | 93.3% |
|  |  |  | **Accuracy** | 69.2% | 69.8% | 69.8% | 67.4% | 67.4% | 68.6% |
|  |  |  | **AUROC** | 0.500 | 0.681 | 0.689 | 0.663 | 0.625 | 0.618 |
| **Etanercept** | 0.99 (0.34) | 73/220  (33.2%) | **Sensitivity** | 0.0 | 42.9% | 38.1% | 42.9% | 0.0% | 19.0% |
|  |  |  | **Specificity** | 100.0 | 77.3% | 84.1% | 77.3% | 100.0% | 93.2% |
|  |  |  | **Accuracy** | 66.8% | 65.4% | 67.7% | 64.6% | 67.7% | 67.7% |
|  |  |  | **AUROC** | 0.500 | 0.656 | 0.667 | 0.629 | 0.661 | 0.640 |
| **Golimumab** | 0.97 (0.30) | 40/122  (32.8%) | **Sensitivity** | 0.0 | 0.0% | 33.3% | 50.0% | 41.7% | 0.0% |
|  |  |  | **Specificity** | 100.0 | 100.0% | 83.3% | 70.8% | 87.5% | 100.0% |
|  |  |  | **Accuracy** | 67.2% | 66.7% | 66.7% | 61.1% | 72.2% | 66.7% |
|  |  |  | **AUROC** | 0.500 | 0.688 | 0.698 | 0.632 | 0.687 | 0.621 |
| **Infliximab** | 0.85 (0.32) | 45/162  (27.8%) | **Sensitivity** | 0.0 | 30.8% | 19.2% | 30.8% | 0.0% | 15.4% |
|  |  |  | **Specificity** | 100.0 | 82.9% | 88.6% | 80.0% | 100.0% | 82.9% |
|  |  |  | **Accuracy** | 72.2% | 66.7% | 70.8% | 66.7% | 72.9% | 65.6% |
|  |  |  | **AUROC** | 0.500 | 0.614 | 0.633 | 0.547 | 0.598 | 0.505 |
| **Abatacept** | 0.99 (0.30) | 55/194  (28.4%) | **Sensitivity** | 0.0 | 18.8% | 25.0% | 37.5% | 0.0% | 0.0% |
|  |  |  | **Specificity** | 100.0 | 90.2% | 85.4% | 80.5% | 100.0% | 100.0% |
|  |  |  | **Accuracy** | 71.6% | 70.2% | 68.4% | 68.4% | 71.9% | 71.9% |
|  |  |  | **AUROC** | 0.500 | 0.626 | 0.653 | 0.636 | 0.617 | 0.517 |
| **Tocilizumab** | 1.01 (0.26) | 235/410  (57.3%) | **Sensitivity** | 0.0 | 71.4% | 75.7% | 72.9% | 75.7% | 68.6% |
|  |  |  | **Specificity** | 100.0 | 36.5% | 32.7% | 34.6% | 30.8% | 34.6% |
|  |  |  | **Accuracy** | 57.3% | 57.4% | 57.4% | 56.6% | 56.6% | 55.7% |
|  |  |  | **AUROC** | 0.500 | 0.580 | 0.582 | 0.555 | 0.536 | 0.517 |

Notes: Biologic DMARDs: six drugs including adalimumab, etanercept, golimumab, infliximab, abatacept, and tocilizumab; TNF inhibitors: four drugs such as adalimumab, etanercept, golimumab, infliximab; Non_TNF inhibitors: two drugs such as abatacept and tocilizumab. N: total number of sample for the drug category. Baseline accuracy: Remission rate not achieved by clinicians; Baseline AUROC: the value when selecting random or one side.

**Table S6. SHAP values for variables in Shapely plots.**

| **Ranking** | **All bDMARDs** | **TNF inhibitor** | **Non-TNF inhibitor** | **Adalimumab** | **Etanercept** | **Infliximab** | **Golimumab** | **Abatacept** | **Tocilizumab** |
| --- | --- | --- | --- | --- | --- | --- | --- | --- | --- |
| 1 | ESR  0.119 | ESR  0.209 | ESR  0.365 | Age  0.266 | RF  0.200 | ESR  0.561 | ESR  0.288 | Disease duration  0.118 | CRP  0.107 |
| 2 | DAS28-ESR  0.061 | Hemoglobin  0.165 | CRP  0.349 | Hemoglobin  0.163 | ESR  0.140 | Hemoglobin  0.386 | ACPA  0.086 | Hemoglobin  0.100 | ESR  0.098 |
| 3 | Hemoglobin  0.056 | Disease duration  0.135 | ALT  0.315 | AST  0.093 | Platelet  0.106 | Disease duration  0.309 | DAS28-ESR  0.064 | CRP  0.095 | DAS28-ESR  0.087 |
| 4 | TG  0.044 | DAS28-ESR  0.135 | RF  0.291 | Disease duration  0.068 | DAS28-ESR  0.106 | Glucose  0.281 | Disease duration  0.055 | ESR  0.087 | Platelet  0.087 |
| 5 | Disease duration  0.030 | Age  0.092 | Platelet  0.258 | DAS28-ESR  0.062 | CRP  0.082 | ACPA  0.281 | Platelet  0.054 | DAS28-ESR  0.078 | ALT  0.070 |
| 6 | CRP  0.021 | TG  0.091 | Age  0.230 | ALT  0.058 | Hemoglobin  0.065 | Treatment of LTBI  0.251 | Age  0.045 | Platelet  0.077 | BMI  0.066 |
| 7 | ACPA  0.015 | ACPA  0.077 | DAS28-ESR  0.228 | Cholesterol  0.055 | Cholesterol  0.038 | Cholesterol  0.239 | TG  0.022 | ALT  0.070 | Age  0.056 |
| 8 | Platelet  0.013 | CRP  0.067 | MTX dose  0.222 | ACPA  0.047 | ACPA  0.021 | Age  0.238 | CRP  0.016 | BMI  0.066 | RF  0.049 |
| 9 | Age  0.013 | Cholesterol  0.063 | Hemoglobin  0.178 | RF  0.029 | BUN  0.019 | ALT  0.226 | Hemoglobin  0.012 | BUN  0.054 | TG  0.041 |
| 10 | Cholesterol  0.013 | History of herpes zoster  0.049 | ACPA  0.170 | LDL  0.026 | TG  0.016 | RF  0.190 | RF  0.010 | Age  0.050 | Cholesterol  0.029 |
| 11 | LDL  0.009 | LDL  0.046 | TG  0.148 | ESR  0.024 | MTX dose  0.016 | Prednisolone dose  0.172 | MTX dose  0.007 | RF  0.044 | Prednisolone dose  0.026 |
| 12 | RF  0.008 | RF  0.037 | Prednisolone dose  0.131 | TG  0.022 | Age  0.015 | CRP  0.127 | Sex  0.006 | TG  0.036 | White blood cell  0.025 |
| 13 | MTX dose  0.005 | Treatment of LTBI  0.034 | ANA  0.127 | MTX dose  0.009 | History of herpes zoster  0.010 | DAS28-ESR  0.117 | ALT  0.006 | Cholesterol  0.029 | History of herpes zoster  0.021 |
| 14 | Treatment of LTBI  0.002 | Lung diseases  0.018 | History of herpes zoster  0.115 | CRP  0.009 | Glucose  0.010 | LDL  0.077 | Prednisolone dose  0.002 | White blood cell  0.025 | ACPA  0.018 |
| 15 | ANA  0.002 | HBs antibody negative  0.014 | History of tuberculosis  0.088 | History of herpes zoster  0.008 | HBs antigen positive  0.004 | ANA  0.060 | Hypercholesterolemia  0.002 | Prednisolone dose  0.023 | MTX dose  0.017 |
| 16 | Lung diseases  0.002 | Sex  0.014 | Fracture or arthroplasty  0.088 | Hypercholesterolemia  0.008 | Treatment of LTBI  0.003 | HBC antibody positive  0.052 | Non-smoker  0.001 | ACPA  0.021 | Non-smoker  0.009 |
| 17 | Hypercholesterolemia  0.001 | MTX dose  0.008 | LDL  0.085 | Prednisolone dose  0.007 | LDL  0.002 | MTX dose  0.046 | Hypertension  0.001 | History of herpes zoster  0.018 | ANA  0.009 |
| 18 | Fracture or arthroplasty  0.001 | Hypercholesterolemia  0.007 | HDL  0.072 | Fracture or arthroplasty  0.005 | Prednisolone dose  0.002 | Sex  0.015 | Liver diseases  <0.001 | MTX dose  0.017 | LDL  0.009 |
| 19 | History of tuberculosis  0.001 | Prednisolone dose  0/005 | Non-smoker  0.042 | Sex  0.005 | IGRA  0.002 | Hypertension  0.012 | Neurological disorder  <0.001 | History of tuberculosis  0.014 | IGRA  0.007 |
| 20 | Prednisolone dose  0.001 | ANA  0.004 | Hypertension  0.035 | ANA  0.005 | HDL  0.001 | HBs antibody negative  0.010 | HBC antibody positive  <0.001 | LDL  0.009 | HDL  0.007 |
| 21 | Sex  0.001 | IGRA  0.003 | HBs antigen positive  0.030 | Diabetes mellitus  0.005 | Lung diseases  0.001 | Non-smoker  0.007 | IGRA  <0.001 | ANA  0.008 | Fracture or arthroplasty  0.007 |
| 22 | IGRA  0.001 | Diabetes mellitus  0.003 | Hypercholesterolemia  0.022 | Non-smoker  0.003 | Tuberculin skin test  0.001 | History of tuberculosis  0.003 | Treatment of LTBI  <0.001 | Non-smoker  0.008 | Hypercholesterolemia  0.003 |
| 23 | Hypertension  <0.001 | HBC antibody positive  0.002 | HBs antibody negative  0.022 | Cardiovascular diseases  0.002 | Sex  0.001 | Drug addition  <0.001 | Gastroduodenal ulcer  <0.001 | IGRA  0.007 | HBs antigen positive  0.002 |
| 24 | Non-smoker  <0.001 | Thyroid diseases  0.001 | Lung diseases  0.020 | HBs antibody  Negative  0.002 | ANA  <0.001 | Cardiovascular diseases  <0.001 | Tuberculin skin test  <0.001 | Fracture or arthroplasty  0.007 | HBs antibody negative  0.001 |
| 25 | HBC antibody positive  <0.001 | Non-smoker  0.001 | Sex  0.010 | Treatment of LTBI  0.002 | HBs antibody negative  <0.001 | Neurological disorder  <0.001 | Diabetes mellitus  <0.001 | HDL  0.007 | Sex  0.001 |
| 26 | HBs antibody negative  <0.001 | Hypertension  0.001 | Tuberculin skin test  0.007 | IGRA  0.002 | HBC antibody positive  <0.001 | Lung diseases  <0.001 | History of herpes zoster  <0.001 | Hypercholesterolemia  0.003 | Neurological disorder  0.001 |
| 27 | HBs antigen positive  <0.001 | HBs antigen positive  0.001 | Neurological disorder  0.002 | HBC antibody positive  0.001 | Gastroduodenal ulcer  <0.001 | Psychiatric problem  <0.001 | Cardiovascular diseases  <0.001 | Hypertension  0.003 | Cardiovascular diseases  <0.001 |
| 28 | Psychiatric problem  <0.001 | Drug addiction  <0.001 | HBC antibody positive  0.002 | Gastroduodenal ulcer  <0.001 | Thyroid diseases  <0.001 | HBs antigen positive  <0.001 | HBs antigen positive  <0.001 | Lung diseases  0.002 | HCV positive  <0.001 |
| 29 | Tuberculin skin test  <0.001 | HCV positive  <0.001 | HCV positive  <0.001 | Lung diseases  <0.001 | Neurological disorder  <0.001 | HCV positive  <0.001 | HCV positive  <0.001 | HBs antigen positive  0.002 | HBC antibody positive  <0.001 |
| 30 | Drug addiction  <0.001 |  |  | HBs antigen positive  <0.001 | HCV positive  <0.001 |  | HBs antibody negative  <0.001 | HBs antibody negative  0.002 |  |
| 31 | HCV positive  <0.001 |  |  | HCV positive  <0.001 | Liver diseases  <0.001 |  | ANA  <0.001 | Neurological disorder  0.001 |  |
| 32 |  |  |  |  | Non-smoker  <0.001 |  |  | Sex  0.001 |  |

ACPA, anti-CCP antibody; ALT, alanine aminotransferase; ANA, anti-nuclear antibody; AST, aspartate aminotransferase; HBsAg, Hepatitis B surface antigen; HBsAb, Hepatitis B surface antibody; HBcAb, Hepatitis B core antibody; HCV, hepatitis C virus; BUN, blood urea nitrogen; CRP, C-reactive protein; DAS28-ESR, disease activity scores in 28 joints using the erythrocyte sedimentation rate; ESR, erythrocyte sedimentation rate; HDL, high-density lipoprotein; IGRA, interferon-gamma release assay; LDL, low-density lipoprotein; LTBI, latent tuberculosis infection; MTX, methotrexate; RF, rheumatoid factor; TG; triglyceride.

**Table S7. Order of important features for prediction of remission without increasing prednisolone dose in each bDMARD.**

| **Clinical variable** | **Average ranking** | **Adalimumab** | **Etanercept** | **Infliximab** | **Golimumab** | **Abatacept** | **Tocilizumab** |
| --- | --- | --- | --- | --- | --- | --- | --- |
| ESR | 3.50 | -0.024 | -0.140 | -0.561 | -0.288 | -0.087 | -0.097 |
| DAS28.ESR | 5.33 | -0.062 | -0.106 | -0.117 | -0.064 | -0.078 | -0.089 |
| CRP | 7.00 | 0 | +0.082 | +0.127 | 0 | +0.095 | +0.107 |
| Age | 7.17 | -0.266 | 0 | -0.238 | -0.045 | -0.050 | -0.056 |
| Hemoglobin | 8.00 | +0.163 | +0.065 | +0.386 | 0 | +0.100 | 0 |
| Rheumatoid.factor | 8.00 | +0.029 | -0.200 | *0.190 | 0 | -0.044 | -0.049 |
| Anti.CCP.antibody | 8.50 | -0.047 | 0 | -0.268 | -0.086 | 0 | 0 |
| Cholesterol | 10.17 | -0.055 | -0.038 | -0.239 | 0 | -0.029 | -0.029 |
| Disease.duration | 10.67 | -0.068 | 0 | *0.309 | -0.055 | -0.118 | 0 |
| ALT | 10.67 | -0.058 | 0 | +0.226 | 0 | -0.070 | -0.070 |
| Platelet | 11.67 | 0 | -0.106 | 0 | +0.054 | -0.077 | -0.087 |
| Trygliceride | 12.33 | -0.022 | 0 | 0 | 0 | -0.036 | -0.041 |
| methotrexate.dose | 13.67 | 0 | 0 | +0.046 | 0 | 0 | 0 |
| Prednisolone.dose | 13.83 | 0 | 0 | +0.172 | 0 | -0.023 | -0.026 |
| Low.density.lipoprotein | 18.33 | -0.026 | 0 | -0.077 | 0 | 0 | 0 |

Notes: Asterisk denotes a non-linear relationship such as quadratic effect or mixed effect between drugs and variables; The average ranking was obtained by averaging the rankings of the 6 bDMARDs

ESR, erythrocyte sedimentation rate; DAS28-ESR, disease activity scores in 28 joints using the erythrocyte sedimentation rate; CRP, C-reactive protein; ALT, alanine aminotransferase; BUN, blood urea nitrogen; ANA, anti-nuclear antibody.

**Figure S1**. Shapley plots predicted.


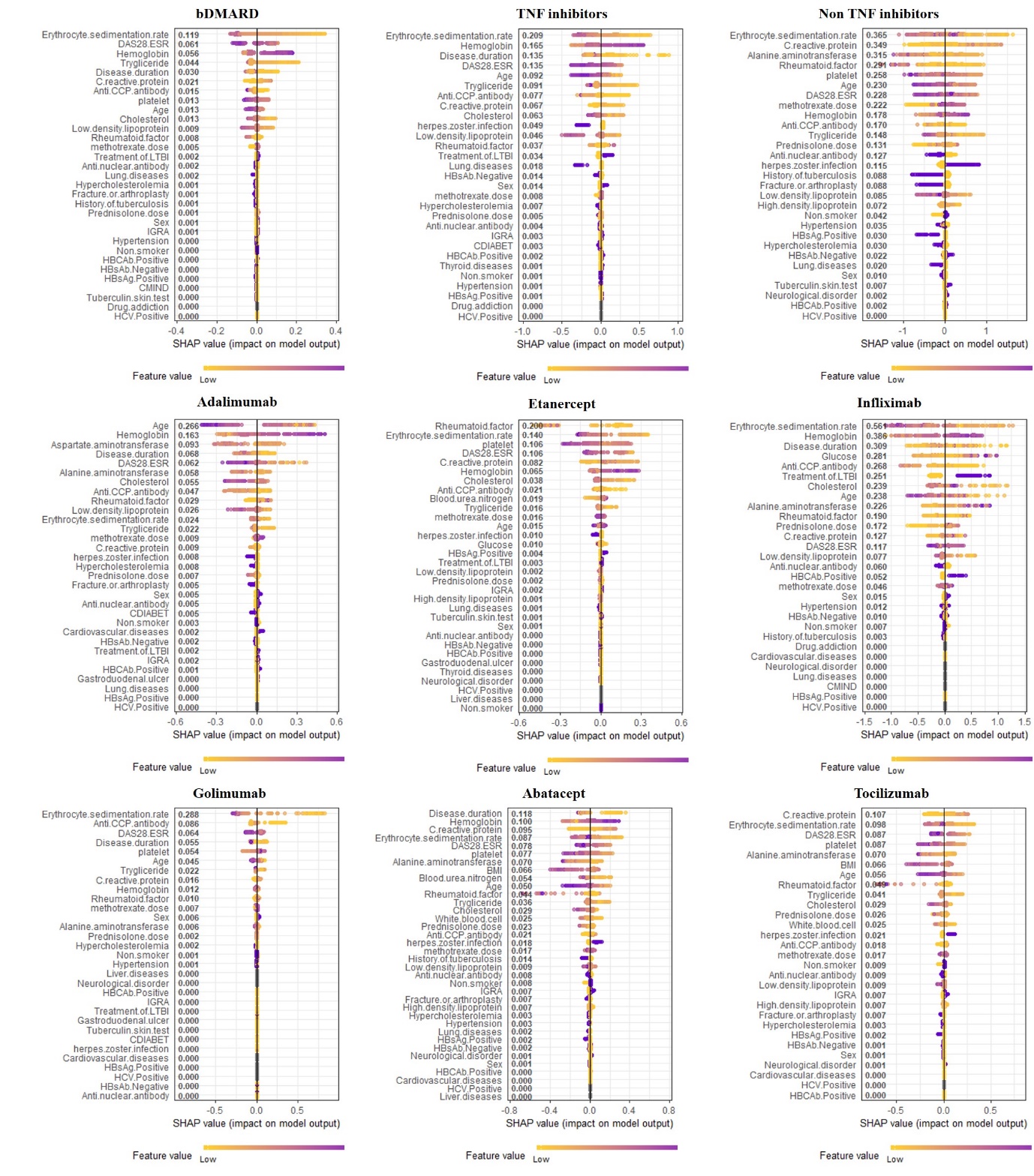


DAS, disease activity score; ESR, erythrocyte sedimentation rate; LTBI, latent tuberculosis infection; IGRA, interferon-gamma release assay; HBcAb, hepatitis B core antibody; CMIND, psychiatric comorbidity; HBsAb, hepatitis B surface antibody; HBsAg, hepatitis B surface antigen; HCV, hepatitis C virus; COSP, osteoporosis; CDIABET, diabetes mellitus and its complication; BMI, body mass index.
